# α-Anilinoketones, Esters and Amides: A Chemical Study

**DOI:** 10.3390/ph5060591

**Published:** 2012-06-05

**Authors:** Amjad M. Qandil, Lara I. Fakhouri

**Affiliations:** 1 Department of Medicinal Chemistry and Pharmacognosy, Faculty of Pharmacy, Jordan University of Science and Technology, Irbid 22110, Jordan; 2 Pharmaceutical Sciences Department, College of Pharmacy, King Saud bin Abdulaziz University for Health Sciences, Riyadh 11426, Saudi Arabia

**Keywords:** α-anilinoketones, 2-anilinoalcohols, α-anilinoesters, α-haloketones, intramolecular hydrogen bond

## Abstract

A group of α-anilinoketones, 2-aminoalcohols, α-anilinoesters and α-anilinoamides were successfully synthesized and characterized by NMR spectroscopy and mass spectrometry. The yields were, in general, moderate to good (up to 75.4%), except for the α-anilinoesters (16.9–35.6%). The α-halocarbonyl starting materials showed different chemical reactivities. α-Haloketones and α-chloroacetates afforded monoalkylation, while small α-chloroamides afforded dialkylation. Finally, NMR spectroscopy revealed interesting structural features about the 2-aminoalcohols and diphenylamides.

## 1. Introduction

**Figure 1:**
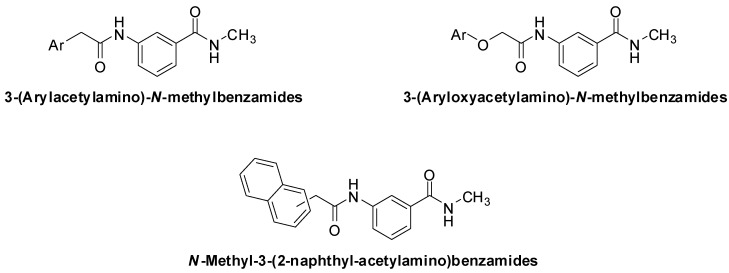
The general structures of 3-(arylacetylamino)-*N*-methylbenzamides, 3-(aryloxyacetylamino)-*N*-methylbenzamides and *N*-methyl-3-(2-naphthyl-acetylamino)-benzamides.

Ando *et al*. have reported a structurally simple group of 3-(arylacetylamino)-*N*-methylbenzamides and 3-(aryloxyacetylamino)-*N*-methylbenzamides (Figure 1) as potent and selective anti-*Helicobacter pylori* agents [1,2]. The naphthylacetylamino derivatives were the most active, with an MIC of 0.1 μg/mL for the 1-naphthyl derivative and 0.05 μg/mL for the 2-naphthyl derivative. The activity of the potent compounds was unaffected by pH changes and they were very selective against *H. pylori*, showing no significant activity against *Staphylococcus aureus*, *Bacillus subtilis*, *Escherichia coli*, *Pseudomonas aeruginosa*, *Bacteroides fragilis* and *Candida albicans* [1,2].

**Figure 2:**
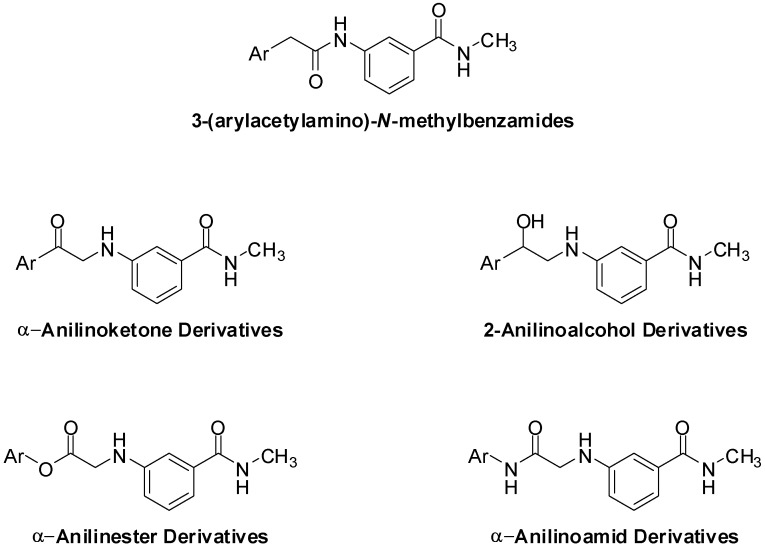
The chemical structures of the target α-anilinoketone, 2-anilinoalcohol, α-anilinoamides and α-anilinoester derivatives.

*H. pylori* is a wide spread pathogen, especially in developing countries, where the incidence of infection can reach up to 50% of the entire population [3]. Infection with *H. pylori* has been directly associated with the development of duodenal ulcers; 95% of patients reporting to clinics with duodenal ulcers are infected with *H. pylori* [4,5]. There is also an accumulating body of evidence that associates *H. pylori* infection the occurrence of stomach cancer, the 2nd most common type of cancer [6,7]. Furthermore, *H. pylori* has also been associated with some types of lymphomas [8], hepatic encephalopathy [9,10] and ischemic heart diseases [11]. The simplicity and selectivity of Ando *et al.*’s compounds have prompted us to design and synthesize a structurally analogous series of α-anilinocarbonyls (ketones, esters and amide, Figure 2).

Herein, the synthesis of these compounds, in addition to the reduction of the ketone derivatives to the corresponding 2-anilinooalchols will be discussed. Also, some interesting spectroscopic details for the synthesized compound and the differences in reactivity of the α-halocarbonyl starting materials will be explained. It is worth mentioning that the antimicrobial activity of these compounds has been determined but will not be published at this time.

## 2. Results and Discussion

### 2.1. Synthesis of α-Anilinoketones ***6***–***15*** and 2-Aminoalchols ***16***–***23***

**Scheme 1:**
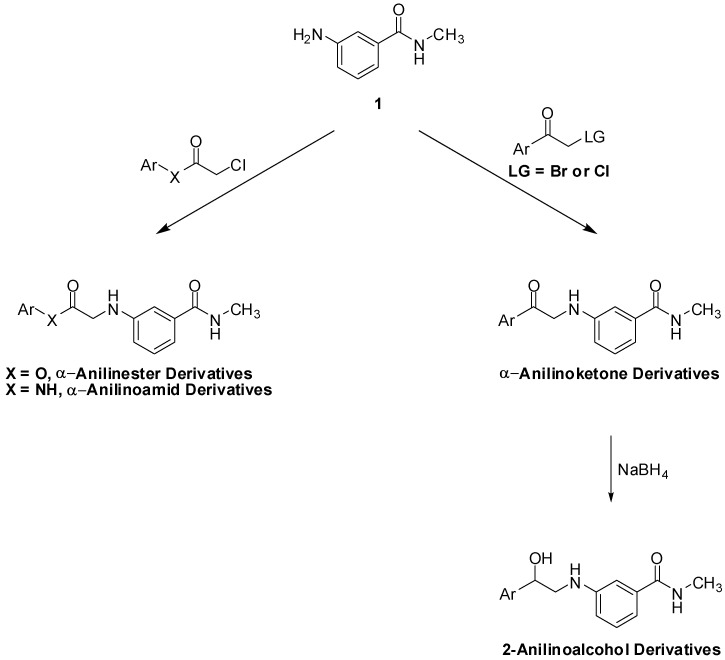
General scheme for the synthesis of target compounds.

Our general synthetic route involved coupling of 3-amino-*N*-methylbenzamide (**1**) to the appropriate α-haloketone, α-haloamide or α-haloester, in DMF in the presence of an inorganic base (Scheme 1). The α-anilinoketones, were further reduced to the corresponding 2-anilinoaclohols using sodium borohydride.

**Scheme 2:**
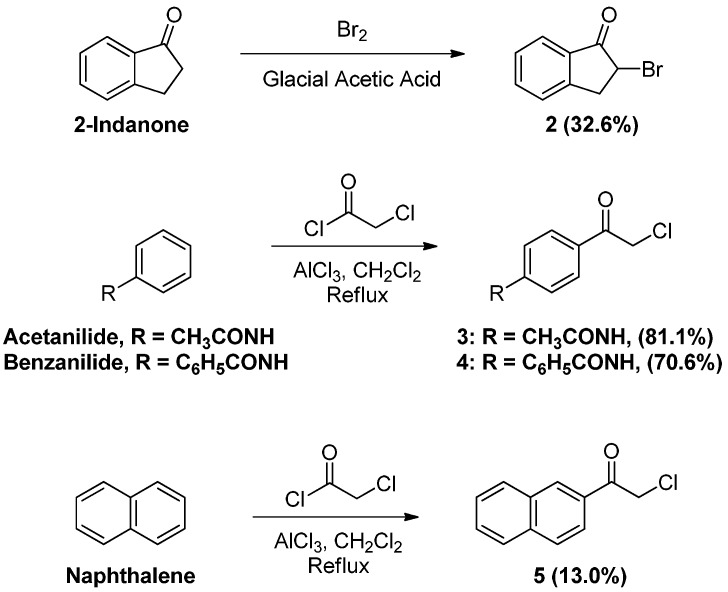
Synthesis of α-bromo and α-chloroketones **2**–**5**.

While many phenacylbromides and chlorides are commercially available, some were prepared for the purpose of this work (Scheme 2). 2-Bromoindan-1-one (**2**) was obtained in low yields by treating 1-indanone with bromine in glacial acetic acid [12,13]. Phenacylchorides **3** and **4**, on the other hand, were prepared by modifying the procedure of Leiserson and Weissberger for the large scale synthesis of *p*-(α-chloroacetyl)acetanilide [14]. Friedel-Crafts acylation of acetanilide and benzanilide with α-chloroacetyl chloride was performed in dichloromethane, instead of carbon disulfide, in the presence of aluminum chloride to afford *p*-(α-chloroacetyl)acetanilide (**3**) and *p*-(α-chloroacetyl)benzanilide (**4**) in good yields. Naphthalene was regioselectively acylated at C-2 to form 2-naphthacylchloride (**5**), confirmed by singlet at 8.49 corresponding to the proton at C-1 of the naphthalene ring in its ^1^H-NMR spectrum.

**Scheme 3:**
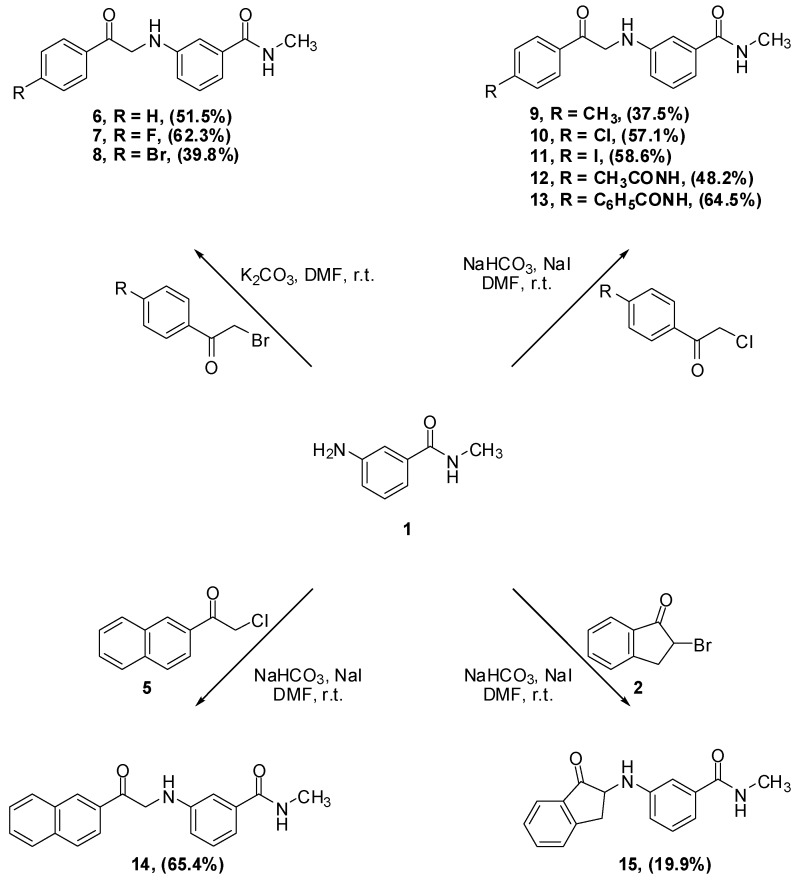
Synthesis of α-aminoketone derivatives **6**–**15**.

The synthesis of α-anilinoketones **6**–**25** is illustrated in Scheme 3. Reaction of phenacylbromide, 4-fluorophenacylbromide and 4-bromophenacylbromide with aniline **1** was straightforward, leading, rather surprisingly, to monoalkylation to afford anilinoketones **6**, **7** and **8**, respectively.

**Scheme 4:**
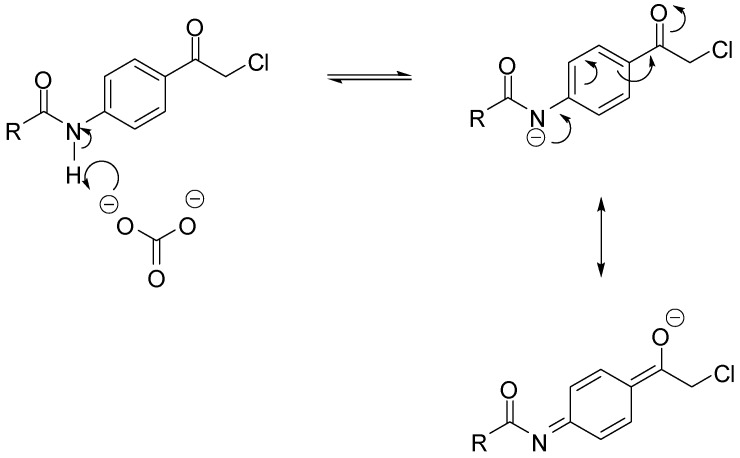
Resonance stabilization of the conjugate base of *p*-(α-chloroacetyl)acylanilides **12** and **13**.

The work up consisted of adding water to the reaction mixtures, which caused immediate precipitation of the crude product. The filtered solid was then crystallized from the appropriate solvent to afford the desired compound. As for alkylation with α-chloroketones, two modifications were found to be necessary. The first was the addition of sodium iodide to facilitate the otherwise sluggish reaction [15]. The iodine atom displaces the α-chlorine producing *in situ* a more electrophilic α-iodoketone [16]. The second modification was replacing potassium carbonate by the less basic sodium bicarbonate because coupling of acetanilide **3** to aniline **1** in the presence of potassium carbonate failed to afford the expected product. It was postulated that potassium carbonate was strong enough to deprotonate the anilide and hence, decreased its nucleophilicity. The amide in **3** is expected to have enhanced acidity due to the fact that the formed conjugate base is efficiently stabilized by resonance, as shown in Scheme 4. Furthermore, the reaction of aniline **1** with 2-bromoindan-1-one **2** in the presence of potassium carbonate afforded multiple products, while the reaction in the presence of sodium bicarbonate instead, was much cleaner, affording 15, albeit in low yield.

**Scheme 5:**
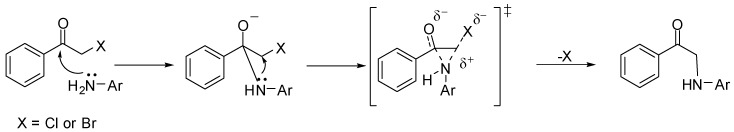
A mechanism involving a bridged-transition state for the alkylation of aniline **1** with α-haloketones.

The exclusive formation of monoalkylated products from the reaction of α-haloketones with aniline **1** can be attributed to an indirect alkylation mechanism, which involves the attack by the amino group in **1** on the carbonyl carbon of the α-haloketones, followed by intramolecular replacement of the halogen, as illustrated in Scheme 5. This mechanism most likely involves a bridged transition state that is easily formed by a primary amine; hence, the produced α-anilinoketones, which have a secondary amine, will not participate in such a mechanism [17]. Furthermore, the bulkier α-anilinoketone, compared to the starting aniline **1**, will have a slim chance to effect a direct second alkylation.

**Scheme 6:**
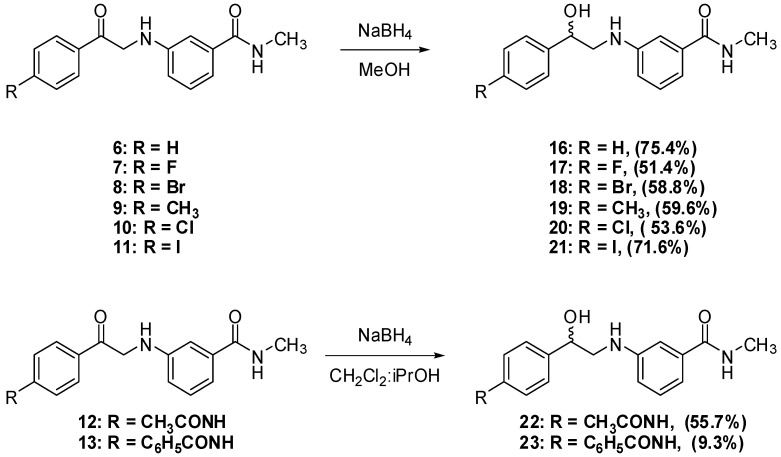
Reduction of α-anilinoketones **6–13** to 2-anilinoalcohols **16–23**.

The reduction of the aromatic ketones **6**–**13** was performed using NaBH_4_. First, the van Vliet *et al.* procedure to reduce α-aminoketones to 2-aminoalcohols was followed. Using sodium borohydride (1.5 equivalents) suspended in a 1:1 dichloromethane-isopropanol mixture, followed by work up under relatively strong acidic conditions [18] reduced compounds **6**, **8** and **12** with satisfactory yields and reduced compound **13** in 9% yield. Alternatively, compounds **7**–**11** were reduced using NaBH_4_ in aqueous basic media. This reaction was generally done by suspending the α-anilinoketone in water, followed by addition of one equivalent of sodium hydroxide and one equivalent of sodium borohydride and the mixture was then stirred overnight. A simple work up by filtering the suspension, then crystallizing the dried cake from ethyl acetate afforded the 2-aminoalcohol. Unfortunately, the yields from this milder and simpler procedure were generally low and α-anilinoketones **7** and **11** failed afford the corresponding 2-anilinoalcohol. Finally, a more standard reduction procedure was employed in which the α-anilinoketones were reduced using one equivalent of sodium borohydride in methanol [19,20]. The yields were higher than the other two procedures and α-anilinoketones **6–11** were reduced to the corresponding 2-aminoaclohols **16–21** in 51.4–75.4% yields. The reduction reactions are summarized in Scheme 6.

**Figure 3:**
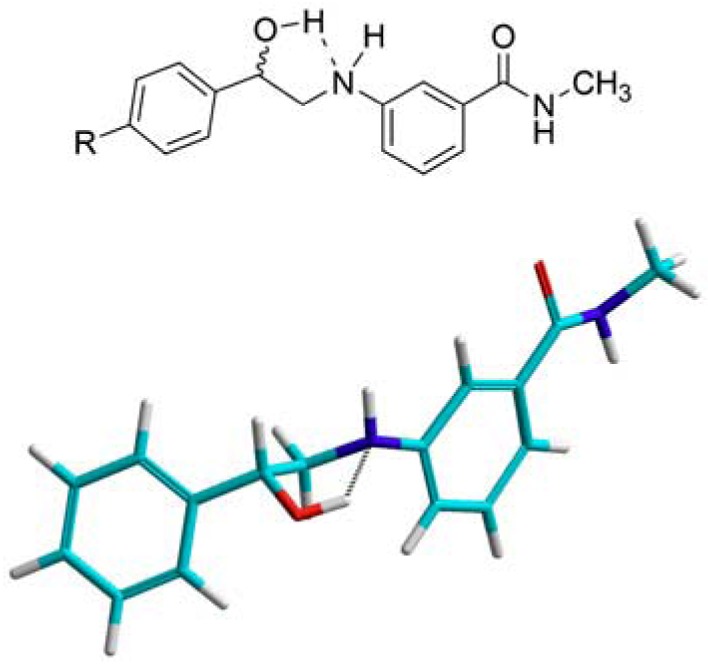
A two-dimensional drawing and a three-dimensional model of a 2-anilinoalcohol showing the suggested intramolecular hydrogen bonding.

Analysis of the ^1^H-NMR spectra of the 2-aminoalcohols showed that the protons on the carbon adjacent to nitrogen are not equivalent and show-up as two distinct multiplets. The splitting pattern in their corresponding peaks is due to vicinal coupling and geminal coupling to the aniline proton and to the proton on the benzylic carbon bearing the hydroxyl group. Such nonequivalency of vicinal methylene protons is usually observed in rigid systems such as small or relatively inflexible rings [21,22,23]. This led to the conclusion that the 2-aminoalcohols are relatively rigid, most likely due to an intramolecular hydrogen bond between the aniline nitrogen’s lone pairs and the hydroxyl group’s hydrogen. This assumption was supported by *ab Initio* calculation using Hyperchem v7.5 as seen in Figure 3.

### 2.2. Synthesis of α-Anilinoesters ***27***–***29***

**Scheme 7:**
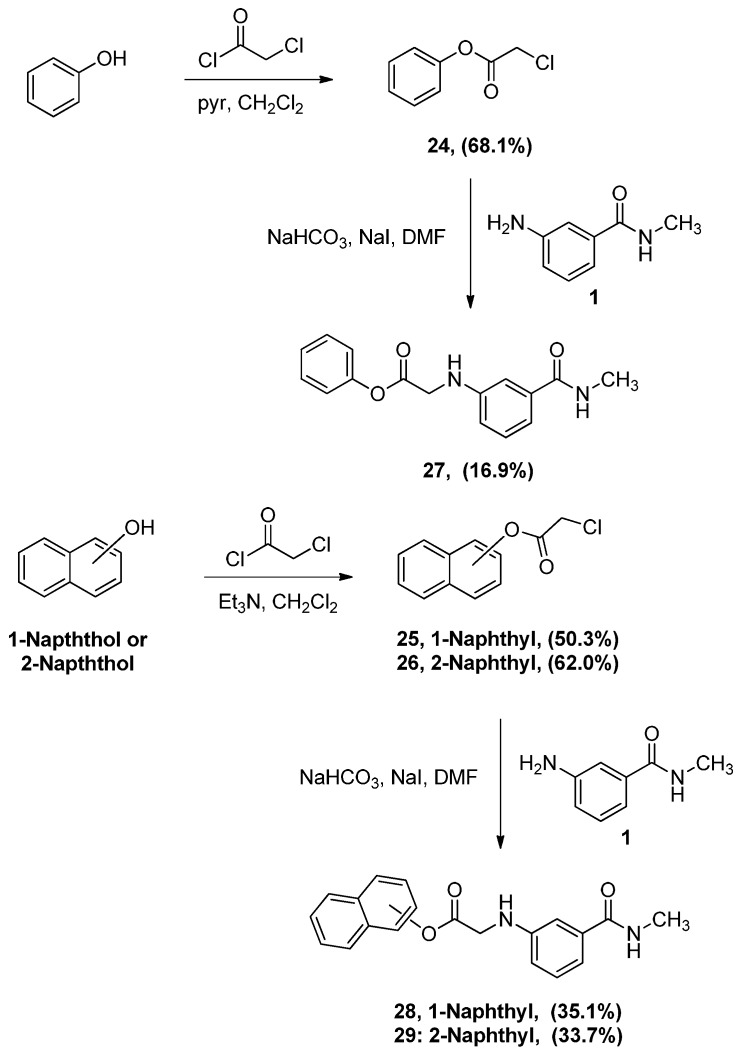
Synthesis of α-anilinoacetates **27-29**.

To prepare the desired aryl-α-anilinoesters, the starting phenyl α-chloroacetate (**24**), 1-naphthyl α-chloroacetate (**25**) and 2-naphthyl α-chloroacetate (**26**) were synthesized by reacting phenol, 1-napththol, and 2-naphthol with α-chloroacetylchloride respectively [24] (Scheme 7).

**Scheme 8:**
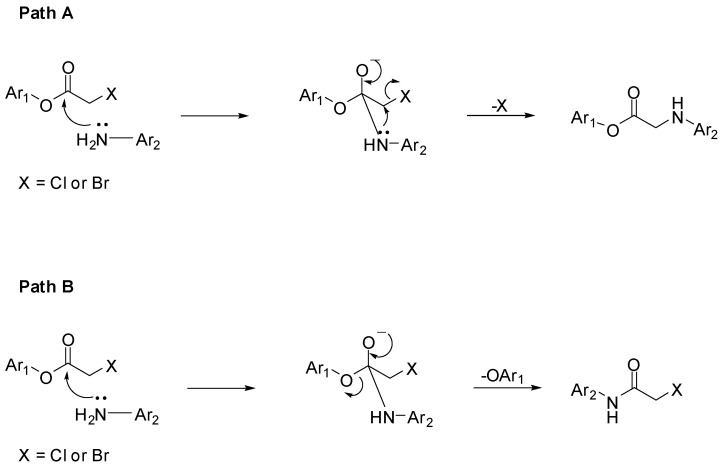
A possible pathway for the decomposition of the tetrahedral intermediate resulting from the reaction of aniline with aryl α-chloroacetates.

The reaction of both naphthols proceeded smoothly in the presence of triethylamine, albeit in moderate yields, but the reaction with phenol using the same conditions yielded more than one product. When pyridine, a weaker base than triethylamine, was used the desired ester **24** was obtained exclusively. Coupling of chloroacetyl esters **24–26** with *N-*methyl-3-aminobenzamide (**1**) was performed following the conditions described earlier as illustrated in Scheme 7. As seen in this scheme, the yields of the α-anilinoacetates **27–29** were rather low. Actually this might indicate that these compounds are formed by the same mechanism of alkylation discussed earlier (Scheme 5). The low yield might be due to the fact that the ester carbonyl’s carbon is less nucleophilic than the ketone’s. Alternatively, the attack of the amino group in aniline **1** on the ester’s carbonyl will lead to the formation of a tetrahedral intermediate as shown in Scheme 8. This tetrahedral intermediate will either proceed to form the desired alkylated aniline (Path **A**) or the aryloxy group will leave leading to the aminolysis (Path **B**). Possible products other than the desired α-anilinoacetates were not investigated.

### 2.3. Synthesis of α-Anilinoesters ***32*** and ***33***

**Scheme 9:**
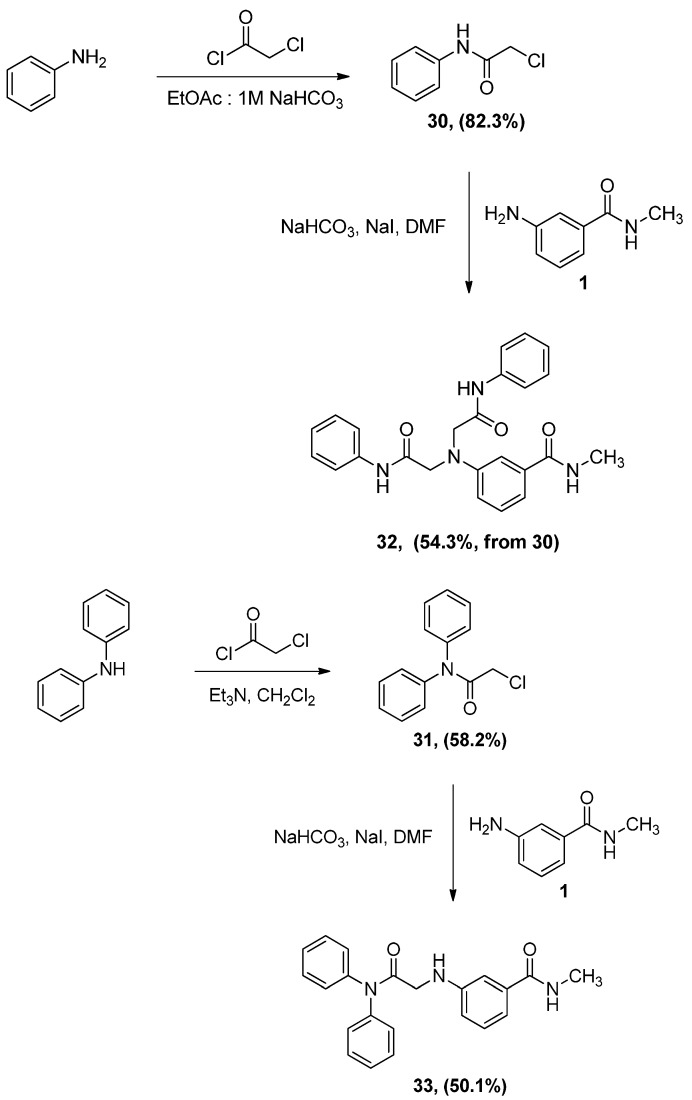
Synthesis of α-aminoacetamides **32** and **33**.

Aniline or diphenylamine were treated with α-chloroacetyl chloride to produce *N*-phenyl α-chloroacetamide (**30**) and *N*,*N*-diphenyl α-chloroacetamide (**31**), respectively. Aniline was coupled to α-chloroacetyl chloride in a biphasic mixture of ethyl acetate and 1 M aqueous sodium bicarbonate to afford amide **30** in very good yield, a procedure that has been used successfully in our labs for chloroacylation of anilines. Alternatively, the stronger triethylamine was used in the prepare *N*,*N*-diphenyl-α-chloroacetamide (**31**). The resultant α-chloroacetamides were then reacted with aniline **1** in the presence of sodium iodide and sodium bicarbonate to afford the α-anilinoester **32** and **33**, as seen in Scheme 9. It was interesting to discover that the ^1^H-NMR and mass spectrum of **32** show, clearly, that two molecules of phenyl α-chloroacetamide **30** have reacted with one molecule of aniline **1** resulting in a dialkylated product. As mentioned earlier such dialkylation was not observed with either α-chloro-ketones or α-chloroacetates.

**Figure 4:**
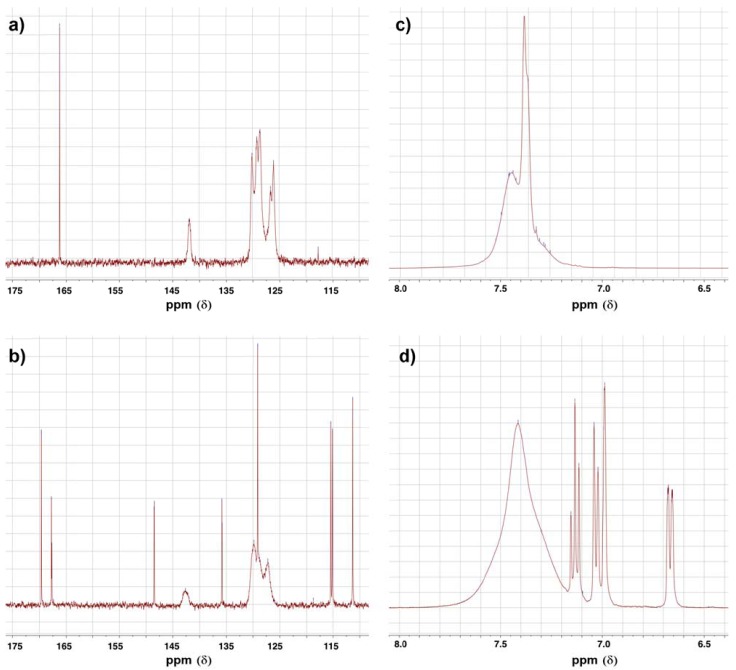
The ^13^C-NMR spectra of (**a**) α-chloroacetamide **31**, (**b**) α-anilinoacetamide **33** and the ^1^H-NMR of (**c**) α-chloroacetamide **31** and (**d**) α-anilinoacetamide **33**.

Alkylation of primary amines by α-chloroacetamides under these experimental conditions was reported earlier to proceed by dialkylation [15], which indicates that this reaction follows an S_N_2 substitution mechanism. This suggestion is supported further by the fact that the bulkier α-chloroacetamide **31** gave the monoalkylation product exclusively, when reacted with aniline **1** in the same conditions. An intriguing aspect of compound **33** and its starting material, α-chloroacetamide **31** was their NMR spectra, specially the ^13^C-NMR spectra. As seen in Figure 4a,b, the peaks corresponding to the carbons in the two phenyl group in the amide side chain of both compounds are not the ordinary sharp narrow peaks usually observed in ^13^C-NMR. These two phenyl groups are slowly rotating as a result of restricted rotation around the amide bond and the N-C_Ph_ bonds, which have partial double bond character. This slowed rotation results in efficient relaxation leading to the broadening observed in these NMR spectra [25]. Here, the two phenyl groups are not equivalent, but not totally distinct enough to appear as two resolved sets of peaks, instead, the peaks were rather wide and unresolved.

## 3. Experimental

### 3.1. Material

Bulk solvents were purchased through local vendors. Reagent grade and fine chemicals were obtained mainly from Aldrich Chemical Company (St. Louis, MO, USA), ACROS Chemicals (Geel, Belgium) and Scharlau Chemical (Barcelona, Spain). Melting points were determined using Stuart Scientific-melting point apparatus (Staffordshire, UK) and are uncorrected. Electron impact (EI) mass spectra were obtained using Shimadzu QP5050A GS-MS (Kyoto, Japan). IR spectra were recorded on Nicolet Avatar 360 FT-IR (Madison, WI, USA) using KBr disks and absorptions are reported in cm^−1^. ^1^H-NMR (400 MHz) and ^13^C-NMR spectra (100 MHz) were acquired using a Bruker Avance Ultrashield instrument (Zürich, Switzerland) and are reported in ppm relative to automatic calibration to the residual proton peak of the solvent used, namely CDCl_3_ or DMSO-*d*_6_. TLC analysis was performed on Albet Silica 60, UV_254_ aluminum-backed TLC plates (Barcelona, Spain). The identities of compounds reported previously in the literature were confirmed by their ^1^H-NMR and melting point data.

### 3.2. Synthesis

*2-Bromoindan-1-one* (**2**). To a solution of 1-indanone (2.0 g, 15.1 mmol) dissolved in glacial acetic acid (10.0 mL), bromine (1.00 mL, 19.50 mmol) was added dropwise and the mixture was stirred for 30 min. The reaction mixture was then dissolved in ether (100 mL) and washed with water (100 mL × 2) and saturated sodium bicarbonate solution (100 mL). The ether layer was then dried (MgSO_4_), filtered and evaporated. The residue was crystallized from ethyl acetate-hexane to yield 1.0 g of the title compound (32.6 %) as white crystals, mp: 41–44 °C, (published: 39–44 °C [26]);^ 1^H-NMR (CDCl_3_): δ 3.43 (dd, 1H, *J* = 18.1, 3.2 Hz); 3.84 (dd, 2H, *J* = 18.1, 7.5 Hz); 4.66 (dd, 2H, CO-CH-Br, *J* = 7.6, 3.2 Hz); 7.49 (m, Ar-H, 2H); 7.72 (t, 2H, Ar-H, *J* = 7.7 Hz); 7.84 (d, 2H, Ar-H, *J* = 7.7 Hz).

*p-Chloroacetyl acetanilide* (**3**). To a solution of acetanilide (20.0 g, 148.0 mmol) and α-chloroacetyl chloride (27.0 mL, 339.5 mmol) in CH_2_Cl_2_ (100 mL), aluminum chloride (59.2 g, 444.0 mmol) was added in portions while stirring, turning the color to dark brown. After completion of addition, the mixture was heated at reflux for 30 min. The reaction mixture was then allowed to cool to room temperature and added slowly with stirring to crushed ice (300 g) containing conc. HCl (10 mL). The precipitate was vacuum filtered, washed thoroughly with water, then left to dry. The solid residue was crystallized from absolute ethanol to yield 25.4 g of **3** (81.1%) as brownish crystals, mp: 219–221 °C (published: 213–214 °C [14]); ^1^H-NMR (DMSO-*d_6_*): δ 2.09 (s, COCH_3_, 3H); 5.09 (s, 2H, CO-CH_2_-Cl, *J* = 18.1, 3.2 Hz) 7.73 (d, 2H, Ar-H, *J* = 8.6 Hz); 7.93 (d, 2H, Ar-H, *J* = 8.5 Hz); 10.34 (s, NH, 1H).

*p-Chloroacetyl benzanilide* (**4**). To a suspension of benzanilide (20.0 g, 101.4 mmol) and α-chloroacetyl chloride (16.0 mL, 201.2 mmol) in CH_2_Cl_2_ (100 mL), aluminum chloride (40.6 g, 304.5 mmol) was added in portions while stirring, turning the color to dark brown. After completion of addition, the mixture was heated at reflux for 2 h. The reaction mixture was then allowed to cool to room temperature and added slowly with stirring to crushed ice (300 g) containing conc. HCl (10 mL). The pale yellow precipitate was vacuum filtered, washed thoroughly with water, then left to dry. The solid residue was crystallized from absolute ethanol to yield 19.6 g of **4** (70.6%) as yellowish-white crystals, mp: 188–191 °C; IR (KBr): 3,353, 1,660, 1,519 cm^−1^; ^1^H-NMR (DMSO-*d_6_*): δ 5.15 (s, 2H, CO-CH_2_-Cl); 7.56 (t, 2H, Ar-H, *J* = 7.4 Hz); 7.63 (t, 1H, Ar-H, *J* = 7.2 Hz); 7.98 (m, 7H, Ar-H); 10.64 (s, 1H, Ar-H, CO-NH). ^13^C-NMR (CDCl_3_): δ 189.81, 165.80, 143.65, 134.27, 132.46, 130.17, 130.02, 129.01, 127.09, 11.92, 54.82, 29.71; MS (EI) *m/z*: 273 (M^+^, 60%); 105 (C_7_H_5_O, 100%).

*2-Naphthacyl chloride* (**5**). To a solution of naphthalene (5.0 g, 39.0 mmol) and α-chloroacetyl chloride (6.2 mL, 78.0 mmol) in CH_2_Cl_2_ (30 mL), aluminum chloride (10.4 g, 78.0 mmol) was added in portions while stirring, turning the color to dark brown. After completion of addition, the mixture was heated at reflux for 30 min. The reaction mixture was then allowed to cool to room temperature and added slowly with stirring to crushed ice (300 g) containing conc. HCl (10 mL). The organic layer was taken, dried (MgSO_4_) and evaporated. The residue was crystallized from ethyl acetate-hexane to yield 2.0 g of **5** (13.0%) as yellow crystals, mp: 115–118 °C, (published: 119–120 °C [27]); IR (KBr): 3,360, 1,675, 1,600 cm^−1^; ^1^H-NMR (CDCl_3_): δ 4.46 (s, 2H, CO-CH_2_-Cl); 7.60 (t, 1H, Ar-H, *J* = 8 Hz); 7.66 (t, 1H, Ar-H, *J* = 8 Hz); 7.91 (d, 1H, Ar-H, *J* = 8 Hz); 7.95 (d, 1H, Ar-H, *J* = 8 Hz); 8.00 (d, 1H, Ar-H, *J* = 8 Hz); 8.01 (dd, 1H, Ar-H, *J* = 2, 8 Hz); 8.49 (s, 1H, Ar-H). MS (EI) *m/z*: 204 (M^+^, 12%), 63 (COCl, 100%).

*N-Methyl-N-3-(2-Phenyl-2-oxo-ethylamino)benzamide* (**6**). Aniline **1** (10.0 g, 66.6 mmol) and phenacyl bromide (13.3 g, 66.6 mmol) were dissolved in DMF (330 mL). To that solution potassium carbonate (9.2 g, 66.6 mmol) was added and the mixture was stirred overnight. The reaction mixture was then added to water (1.2 L) and the precipitate was vacuum filtered and left to dry. The solid residue was crystallized from ethyl acetate to yield 9.2 g of **6** (51.5%) as yellow crystals, mp: 177–179 °C; IR: 3,371, 1,691, 1,515, 1,358 cm^−1^; ^1^H-NMR (DMSO-*d_6_*): δ 2.73 (d, 3H, N-CH_3_, *J* = 4.8 Hz); 4.71 (d, 2H, CO-CH_2_-N-Ar, *J* = 4 Hz); 6.01 (t, 1H, Ar-NH, *J* = 5.2 Hz); 6.81 (dd, 1H, Ar-H, *J* = 8, 2 Hz); 7.00 (d, 1H, Ar-H, *J* = 7.6 Hz); 7.07 (s, 1H, Ar-H); 7.13 (t, 1H, Ar-H, *J* = 7.8 Hz); 7.56 (t, 2H, Ar-H, *J* = 7.6 Hz); 7.66 (t, 1H, Ar-H, *J* = 7 Hz); 8.06 (d, 2H, Ar-H, *J* = 8 Hz); 8.19 (d, 1H, CO-NH, *J* = 4.4 Hz). ^13^C-NMR (DMSO-*d_6_*): δ 196.96, 167.76, 18.60, 135.83, 135.51, 134.04, 29.28, 129.11, 128.31, 115; MS (EI) *m/z*: 268 (M^+^, 42%); 163 (C_9_H_11_N_2_O, 100%).

*N-3-[2-(4-Flourophenyl)-2-oxo-ethylamino]-N-methylbenzamide* (**7**). Aniline **1** (1.0 g, 6.7 mmol) and 4-fluorophenacyl bromide (1.4 g, 6.7 mmol) were dissolved in DMF (15 mL). To that solution, potassium carbonate (0.9 g, 6.5 mmol) was added and the mixture was stirred for 4 h. The reaction mixture was then added to water (200 mL) and the precipitate was vacuum filtered and left to dry. The solid residue was crystallized from absolute ethanol to yield 1.2 g of **7** (62.3%) as yellow crystals, mp: 194–197 °C; IR: 3,381, 3,319, 1,685, 1,600 cm^−1^; ^1^H-NMR (DMSO-*d_6_*): δ 2.73 (d, 3H, N-CH_3_, *J* = 4.5 Hz); 4.70 (d, 2H, CO-CH_2_-N-Ar, *J* = 5.5 Hz); 6.02 (t, 1H, Ar-NH, *J* = 5.4 Hz); 6.81 (dd, 1H, Ar-H, *J* = 8, 2.3 Hz); 7.00 (d, 1H, Ar-H, *J* = 7.5 Hz); 7.07 (s, 1H, Ar-H); 7.12 (t, 1H, Ar-H, *J* = 7.8 Hz); 7.39 (t, 2H, Ar-H, *J* = 7.9 Hz); 8.15 (m, 3H, Ar-H and CO-NH); ^13^C-NMR (DMSO-*d_6_*): δ 195.28, 167.36, 165.16 (C-F, *J* = 250 Hz), 148.18, 135.45, 131.89, 130.98 (C-F, *J* = 9 Hz), 128.70, 116.90 (C-F, *J* = 21 Hz), 115.54 (C-F, *J* = 45 Hz), 110.86, 49.79, 26.26; MS (EI) *m/z*: 286 (M^+^, 42%); 163 (C_9_H_11_N_2_O, 100%).

*N-3-[2-(4-Bromophenyl)-2-oxo-ethylamino]*-*N-methylbenzamide* (**8**). Aniline **1** (5.0 g, 33.3 mmol) and 4-bromophenacyl bromide (9.3 g, 33.3 mmol) were dissolved in DMF (100 mL). To that solution, potassium carbonate (4.6 g, 33.3 mmol) was added and the mixture was stirred for 2 h. The reaction mixture was then added to water (300 mL) and the precipitate was vacuum filtered and left to dry. The solid residue was crystallized from absolute ethanol to yield 4.6 g of **8** (39.8%) as yellow crystals, mp: 196–199 °C; IR: 3,385, 3,311, 1,684, 1,586, 995 cm^−1^; ^1^H-NMR (DMSO-*d_6_*): δ 2.73 (d, 3H, N-CH_3_, *J* = 4.4 Hz); 4.69 (d, 2H, CO-CH_2_-N-Ar, *J* = 5.2 Hz); 6.02 (t, 1H, Ar-NH, *J* = 5.2 Hz); 6.80 (d, 1H, Ar-H, *J* = 8 Hz); 7.00 (d, 1H, Ar-H, *J* = 8 Hz); 7.06 (s, 1H, Ar-H); 7.12 (t, 1H, Ar-H, *J* = 7.8 Hz); 7.77 (d, 2H, Ar-H, *J* = 7.8 Hz); 7.99 (d, 2H, Ar-H, *J* = 7.6 Hz); 8.18 (d, 1H, CO-NH, *J* = 4.4 Hz); ^13^C-NMR (DMSO-*d_6_*): δ 196.33, 167.76, 148.51, 135.83, 134.50, 132.31, 130.34, 129.11, 128.11, 115.71, 115.32, 111.26, 56.51, 26.67; MS (EI) *m/z*: 346 (M^+^, 23%); 163 (C_9_H_11_N_2_O, 100%).

*N-Methyl-N-3-(2-p-tolyl-2-oxo-ethylamino)benzamide* (**9**). To a solution of aniline **1** (3.6 g, 23.6 mmol) and 4-methylphenacy chloride (4.0 g, 23.6 mmol) in DMF (30 mL), sodium bicarbonate (2.0 g, 23.6 mmol) and sodium iodide (5.1 g, 23.6 mmol) were added and the mixture was stirred overnight. The reaction mixture was then added to water (500 mL) and the precipitate was vacuum filtered and left to dry. The solid residue was crystallized from absolute ethanol to yield 2.5 g of **9** (37.5%) as a white powder, mp: 161–163 °C; IR: 3,386, 3,318, 1,688, 1,604, 1,539, 811 cm^−1^; ^1^H-NMR (DMSO-*d_6_*): δ 2.39 (s, 3H, Ar-CH_3_); 2.74 (d, 3H, N-CH_3_, *J* = 4.4 Hz); 4.67 (d, 2H, CO-CH_2_-N-Ar, *J* = 5.2 Hz); 5.98 (t, 1H, Ar-NH, *J* = 5.4 Hz); 6.81 (d, 1H, Ar-H, *J* = 7.6 Hz); 7.00 (d, 1H, Ar-H, *J* = 7.6 Hz); 7.07 (s, 1H, Ar-H); 7.12 (t, 1H, Ar-H, *J* = 7.8 Hz); 7.36 (d, 2H, Ar-H, *J* = 8 Hz); 7.96 (d, 2H, Ar-H, *J* = 8 Hz); 8.19 (d, 1H, CO-NH, *J* = 4.4 Hz); ^13^C-NMR (DMSO-*d_6_*): δ 196.39, 167.76, 148.59, 144.50, 135.83, 133.02, 129.80, 129.09, 128.41, 115.74, 115.23, 111.20, 50.08, 26.66, 21.67; MS (EI) *m/z*: 282 (M^+^, 42%); 163 (C_9_H_11_N_2_O, 100%).

*N-3-[2-(4-Chlorophenyl)-2-oxo-ethylamino]-N-methylbenzamide* (**10**). To a solution of aniline **1** (2.0 g, 13.3 mmol) and 4-chlorophenacyl chloride (2.5 g, 13.4 mmol) in DMF (30 mL), sodium bicarbonate (2.9 g, 20.9 mmol) and sodium iodide (1.1 g, 51.4 mmol) were added and the mixture was stirred overnight. The reaction mixture was then added to water (250 mL) and the precipitate was vacuum filtered and left to dry. The solid residue was crystallized from absolute ethanol to yield 2.4 g of **10** (57.1%) as a white powder, mp: 188–190 °C; IR: 3,386, 3,319, 1,685, 1,612, 997 cm^−1^; ^1^H-NMR (DMSO-*d_6_*): δ 2.73 (d, 3H, N-CH_3_, *J* = 4.4 Hz); 4.70 (d, 2H, CO-CH_2_-N-Ar, *J* = 5.6 Hz); 6.03 (t, 1H, Ar-NH, *J* = 5.4 Hz); 6.81 (dd, 1H, Ar-H, *J* = 2, 8 Hz); 7.00 (d, 1H, Ar-H, *J* = 7.6 Hz); 7.06 (s, 1H, Ar-H); 7.12 (t, 1H, Ar-H, *J* = 7.8 Hz); 7.63 (d, 2H, Ar-H, *J* = 8.4 Hz); 8.07 (d, 2H, Ar-H, *J* = 8.8 Hz); 8.18 (d, 1H, CO-NH, *J* = 4.8 Hz); ^13^C-NMR (DMSO-*d_6_*): δ 200.85, 172.52, 135.27, 143.68, 140.61, 140.57, 138.92, 134.90, 134.10, 135.85, 120.47, 120.08, 116.02, 55.04, 31.28; MS (EI) *m/z*: 302 (M^+^, 37%); 163 (C_9_H_11_N_2_O, 100%).

*N-3-[2-(4-Iodophenyl)-2-oxo-ethylamino]-N-methylbenzamide* (**11**). To a solution of aniline **1** (1.2 g, 8.2 mmol) and 4-iodophenacyl chloride (2.3 g, 8.2 mmol) in DMF (30 mL), sodium bicarbonate (688.8 mg, 8.2 mmol) and sodium iodide (1.8 g, 8.2 mmol) were added and the mixture was stirred overnight. The reaction mixture was then added to water (200 mL) and the precipitate was vacuum filtered and left to dry. The solid residue was crystallized from absolute ethanol to yield 1.9 g of **11** (58.8%) as yellowish crystals, mp: 210–213 °C; IR: 3,388, 1,686, 993 cm^−1^; ^1^H-NMR (DMSO-*d_6_*): δ 2.73 (d, 3H, N-CH_3_, *J* = 4.4 Hz); 4.67 (d, 2H, CO-CH_2_-N-Ar, *J* = 5.6 Hz); 6.01 (t, 1H, Ar-NH, *J* = 5.4 Hz); 6.80 (dd, 1H, Ar-H, *J* = 2, 8 Hz); 7.00 (d, 1H, Ar-H, *J* = 7.6 Hz); 7.06 (s, 1H, Ar-H); 7.12 (t, 1H, Ar-H, *J* = 7.8 Hz); 7.81 (d, 2H, Ar-H, *J* = 8.4 Hz); 7.95 (d, 2H, Ar-H, *J* = 8.4 Hz); 8.18 (d, 1H, CO-NH, *J* = 4.4 Hz); ^13^C-NMR (DMSO-*d_6_*): δ 196.66, 167.73, 167.66, 148.50, 138.19, 135.81, 134.79, 130.04, 129.10, 115.70, 115.30, 111.24, 102.65, 50.19, 26.54; MS (EI) *m/z*: 394 (M^+^, 38%); 163 (C_9_H_11_N_2_O, 100%).

*N-3-[2-(4-Acetylaminophenyl)-2-oxo-ethylamino]-N-methylbenzamide* (**12**). To a solution of aniline **1** (4.5 g, 30.0 mmol) and *p*-chloroacetyl acetanilide (**3**) (6.4 g, 30.2 mmol) in DMF (100 mL), sodium bicarbonate (2.5 g, 29.4 mmol) and sodium iodide (6.5 g, 43.4 mmol) were added and the mixture was stirred overnight. The reaction mixture was then added to water (300 mL) and the precipitate was vacuum filtered and left to dry. The solid residue was crystallized from DMF-water to yield 4.7 g of **12** (48.2%) as yellowish brown crystals, mp: 226–230 °C; IR: 3,375, 3,330, 1,682, 1,598, 1,538 cm^−1^; ^1^H-NMR (DMSO-*d_6_*): δ 2.08 (s, 3H, CH_3_-CO-N); 2.74 (d, 3H, N-CH_3_, *J* = 4.4 Hz); 4.64 (d, 2H, CO-CH_2_-N-Ar, *J* = 5.6 Hz); 5.96 (t, 1H, Ar-NH, *J* = 5.4 Hz); 6.80 (d, 1H, Ar-H, *J* = 7.6 Hz); 6.99 (d, 1H, Ar-H, *J* = 7.6 Hz); 7.06 (s, 1H, Ar-H); 7.12 (t, 1H, Ar-H, *J* = 7.8 Hz); 7.74 (d, 2H, Ar-H, *J* = 8.4 Hz); 8.02 (d, 2H, Ar-H, *J* = 8.8 Hz); 8.19 (d, 1H, CO-NH, *J* = 4.4 Hz); 10.30 (s, 1H, CO-NH-Ar); ^13^C-NMR (DMSO-*d_6_*): δ 195.32, 169.51, 167.80, 148.61, 144.43, 135.84, 130.05, 129.63, 129.09, 118.70, 115.74, 115.20, 111.20, 49.86, 26.67, 24.64; MS (EI) *m/z*: 325 (M^+^, 55%); 163 (C_9_H_11_N_2_O, 100%).

*N-Methyl-N-3-[2-(4-phenacylaminophenyl)-2-oxo-ethylamino]benzamide* (**13**). To a solution of aniline **1** (3.0g, 20.0 mmol) and 4-benzoylaminophenacyl chloride (**4**) (5.5g, 20.0 mmol) in DMF (75 mL), sodium bicarbonate (1.7 g, 20.0 mmol) and sodium iodide (4.3 g, 28. mmol) were added and the mixture was stirred over 72 h. The reaction mixture was then added to water (800 mL) and the precipitate was vacuum filtered and left to dry. The solid residue was crystallized from DMF-water to yield 5.0 g of **13** (64.6%) as yellowish brown crystals; mp: 252–255 °C; IR: 3,406, 3,377, 3,312, 1,676, 1,600, 1,538 cm^−1^; ^1^H-NMR (DMSO-*d_6_*): δ 2.74 (d, 3H, N-CH_3_, *J* = 4.4 Hz); 4.68 (d, 2H, CO-CH_2_-N-Ar, *J* = 5.2 Hz); 5.99 (t, 1H, Ar-NH, *J* = 5.4 Hz); 6.82 (dd, 1H, Ar-H, *J* = 2, 8 Hz); 7.00 (d, 1H, Ar-H, *J* = 7.6 Hz); 7.08 (s, 1H, Ar-H); 7.13 (t, 1H, Ar-H, *J* = 7.8 Hz); 7.55 (t, 2H, Ar-H, *J* = 7.4 Hz); 7.61 (t, 1H, Ar-H, *J* = 7.2 Hz); 7.98 (m, 4H, Ar-H); 8.09 (d, 2H, Ar-H, *J* = 8.8 Hz); 8.20 (d, 1H, CO-NH, *J* = 4.4 Hz); 10.58 (s, 1H, CO-NH-Ar); ^13^C-NMR (DMSO-*d_6_*): δ 195.52, 180.59, 167.81, 167.22, 148.60, 144.41, 135.86, 134.95, 132.42, 130.60, 129.47, 129.11, 128.94, 128.27, 120.01, 119.92, 115.74, 115.23, 111.22, 49.97, 26.67; MS (EI) *m/z*: 387 (M^+^, 8%); 163 (C_9_H_11_N_2_O, 100%).

*N-3-(2-Naphth-2-yl-2-oxo-ethylamino)-N*-*methylbenzamide* (**14**). Aniline **1** (350.0 mg, 2.3 mmol) and 2-naphthacyl chloride (**5**) (0.5 g, 2.4 mmol) were dissolved in DMF (10 mL). To that solution, sodium bicarbonate (193.2 g, 2.3 mmol) of and sodium iodide (480.0 mg, 3.3 mmol) were added and the reaction mixture was stirred overnight. The reaction mixture was then added to water (100 mL) and the precipitate was vacuum filtered and left to dry. The solid residue was crystallized from ethyl acetate to yield 0.5 g of **14** (65.4%) as yellow crystals; mp: 173–175 °C; IR: 3,362, 3,304, 1,683, 1,579 cm^−1^; ^1^H-NMR (DMSO-*d_6_*): δ 2.74 (d, 3H, N-CH_3_, *J* = 4.4 Hz); 4.85 (d, 2H, CO-CH_2_-N-Ar, *J* = 2.7 Hz); 6.08 (s, 1H, Ar-NH); 6.85 (dd, 1H, Ar-H, *J* = 1.6, 8 Hz); 7.01 (d, 1H, Ar-H, *J* = 7.5 Hz); 7.14 (m, 2H, Ar-H); 7.66 (m, 2H, Ar-H); 8.01 (m, 3H, Ar-H); 8.14 (d, 1H, Ar-H, *J* = 7.4 Hz); 8.21 (d, 1H, CO-NH, *J* = 4.4 Hz); ^13^C-NMR (DMSO-*d_6_*): δ 196.92, 167.76, 148.64, 135.89, 135.68, 132.81, 132.64, 130.19, 130.05, 129.24, 129.11, 128.87, 128.20, 127.51, 123.91, 115.75, 115.22, 111.28, 50.27, 26.67; MS (EI) *m/z*: 318 (M^+^, 30%); 163 (C_9_H_11_N_2_O, 100%).

*N-Methyl-N-3-(1-oxo-indan-2-ylamino]benzamide* (**15**). To a solution of aniline **1** (1.0 g, 6.7 mmol) and 2-bromo-indan-1-one (**2**) (1.4 g, 6.7 mmol) in DMF (25 mL), sodium bicarbonate (1.1 g, 13.3 mmol) was added and the reaction mixture was stirred overnight. The reaction mixture was then added to water (300 mL) and the precipitate was vacuum filtered and left to dry. The aqueous layer was extracted with CH_2_Cl_2_ (100 mL × 2) and the organic layer was dried (MgSO_4_), filtered then evaporated. The solid residue combined with the residue from CH_2_Cl_2 _extract was crystallized from ethyl acetate to yield 0.4 g of **15** (19.9 %) as violet crystals, mp: 149–152 °C; IR: 3,368, 3,297, 1,729, 1,638 cm^−1^; ^1^H-NMR (DMSO-*d_6_*): δ 2.73 (d, 3H, N-CH_3_, *J* = 4.4 Hz); 2.89 (dd, 1H, Ar-CH_2_, *J* = 5.2, 16.8 Hz); 3.70 (dd, 1H, Ar-CH_2_, *J* = 8, 16.8 Hz); 4.50 (m, 1H, CO-CH-N-Ar); 6.20 (d, 1H, Ar-NH, *J* = 7.2 Hz); 6.77 (dd, 1H, Ar-H, *J* = 2, 8 Hz); 7.01 (d, 1H, Ar-H, *J* = 7.6 Hz); 7.13 (m, 2H, Ar-H, *J* = 8 Hz); 7.47 (t, 1H, Ar-H, *J* = 7.4 Hz); 7.59 (d, 1H, Ar-H, *J* = 7.6 Hz); 7.71 (t, 2H, Ar-H, *J* = 7 Hz); 8.19 (d, 1H, CO-NH, *J* = 4 Hz); ^13^C-NMR (DMSO-*d_6_*): δ 205.10, 167.73, 151.95, 148.47, 135.94, 135.88, 135.37, 129.22, 128.27, 123.90, 115.64, 115.37, 111.65, 58.79, 34.38, 26.69; MS (EI) *m/z*: 280 (M^+^, 91%); 161 (C_9_H_9_N_2_O, 100%).

*N-3-(2-Hydroxy-2-phenylethylamino)-N-methylbenzamide* (**16**). To a suspension of aminoketone **6** (0.5 g, 1.9 mmol) in methanol (10 mL) at 15 °C, sodium borohydride (71.2 mg, 1.9 mmol) was added, then the mixture was allowed to stir at room temperature till complete dissolution. CH_2_Cl_2_ (20 mL) was then added and the resultant mixture was washed with water (30 mL × 2) followed by 1 M NaOH solution (30 mL × 2). The combined aqueous layers were extracted with CH_2_Cl_2_ (30 mL), and the combined organic layers were dried (MgSO_4_), filtered and evaporated. The residue was crystallized from ethyl acetate-ether to yield 380.0 mg of **16** (75.4%) as white crystals; mp: 116–118 °C; IR: 3,409, 3,328, 1,613, 1,543, 1,333, 694 cm^−1^; ^1^H-NMR (DMSO-*d_6_*): δ 2.75 (d, 3H, N-CH_3_, *J* = 4.5 Hz); 3.15 (m, 1H, CH_2_-N-Ar); 3.26 (m, 1H, CH_2_-N-Ar); 4.76 (m, 1H, Ar-CH); 5.49 (d, 1H, OH, *J* = 4.3 Hz); 5.71 (t, 1H, Ar-NH, *J* = 5.7 Hz); 6.77 (dd, 1H, Ar-H, *J* = 2.2, 8 Hz); 6.99 (d, 1H, Ar-H, *J* = 6.9 Hz); 7.05 (d, 1H, Ar-H, *J* = 1.4 Hz); 7.12 (t, 1H, Ar-H, *J* = 7.2 Hz); 7.26 (t, 1H, Ar-H, *J* = 7.2 Hz); 7.35 (t, 2H, Ar-H, *J* = 7 Hz); 7.42 (d, 2H, Ar-H, *J* = 7.6 Hz); 8.21 (d, 1H, CO-NH, *J* = 4.2 Hz); ^13^C-NMR (DMSO-*d_6_*): δ 167.84, 149.04, 144.58, 135.85, 129.17, 128.63, 127.48, 126.50, 115.29, 114.89, 110.85, 170.24, 51.79, 26.69; MS (EI) *m/z*: 270 (M^+^, 19%); 163 (C_9_H_11_N_2_O, 100%).

*N-3-[2-(4-Flourophenyl)-2-hydroxyethylamino]-N-methylbenzamide* (**17**). To a suspension of the aminoketone **7** (0.3 g, 1.0 mmol) in methanol (8 mL) at 15 °C, sodium borohydride (37.8 mg, 1.0 mmol) was added, then the mixture was allowed to stir at room temperature till complete dissolution. CH_2_Cl_2_ (30 mL) was then added and the resultant mixture was washed with water (30 mL) followed by 1 M NaOH solution (30 mL × 2). The combined aqueous layers were extracted with CH_2_Cl_2_ (30 mL), and the combined organic layers were dried (MgSO_4_), filtered and evaporated. The residue was crystallized from ethyl acetate-ether to yield 0.2 g of **17** (51.3%) as white crystals; mp: 100–102 °C; IR: 3,345, 1,604, 1,340, 1,225, 829 cm^−1^; ^1^H-NMR (DMSO-*d_6_*): δ 2.73 (d, 3H, N-CH_3_, *J* = 4.4 Hz); 3.13 (m, 1H, CH_2_-N-Ar); 3.21 (m, 1H, CH_2_-N-Ar); 4.73 (m, 1H, Ar-CH); 5.52 (d, 1H, OH, *J* = 4 Hz); 5.68 (t, 1H, Ar-NH, *J* = 5.8 Hz); 6.74 (dd, 1H, Ar-H, *J* = 2.4, 8 Hz); 6.96 (d, 1H, Ar-H, *J* = 7.6 Hz); 7.02 (s, 1H, Ar-H); 7.11 (t, 1H, Ar-H, *J* = 7.8 Hz); 7.16 (d, 2H, Ar-H, *J* = 8.6 Hz); 7.42 (m, 2H, Ar-H); 8.19 (d, 1H, CO-NH, *J* = 4.5 Hz); ^13^C-NMR (DMSO-*d_6_*): δ 167.77, 161.95 (C-F, *J* = 240 Hz), 149.04, 140.74, 135.84, 129.13, 128.41 (C-F, *J* = 8 Hz), 115.38 (C-F, *J* = 25 Hz), 114.95 (C-F, *J* = 19 Hz), 110.85, 70.57, 51.65, 26.66; MS (EI) *m/z*: 288 (M^+^, 16%); 163 (C_9_H_11_N_2_O, 100%).

*N-3-[2-(4-Bromophenyl)-2-hydroxyethylamino]-N-methylbenzamide* (**18**). To a suspension of aminoketone **8** (380.0 mg, 1.0 mmol) in methanol (10 mL) at 15 °C, sodium borohydride (45.2 g, 1.2 mmol) was added then the mixture was allowed to stir at room temperature till complete dissolution. CH_2_Cl_2_(20 mL) was then added and the resultant mixture was washed with water (30 mL × 2) followed by 1 M NaOH solution (30 mL × 2). The combined aqueous layers were extracted with CH_2_Cl_2_ (60 mL), and the combined organic layers were dried (MgSO_4_), filtered and evaporated. The residue was crystallized from ethyl acetate-ether to yield 0.2 g of **18** (56.8%) as white crystals, mp: 127–129 °C; IR: 3,443, 3,357, 1,637, 1,604 cm^−1^; ^1^H-NMR (DMSO-*d_6_*): δ 2.73 (d, 3H, N-CH_3_, *J* = 4.4 Hz); 3.15 (m, 1H, CH_2_-N-Ar); 3.20 (m, 1H, CH_2_-N-Ar); 4.73 (m, 1H, Ar-CH); 5.57 (d, 1H, OH, *J* = 4.2 Hz); 5.70 (t, 1H, Ar-NH, *J* = 5.7 Hz); 6.74 (dd, 1H, Ar-H, *J* = 2, 8 Hz); 6.97 (dd, 1H, Ar-H, *J* = 7 Hz); 7.02 (s, 1H, Ar-H); 7.10 (t, 1H, Ar-H, *J* = 7.8 Hz); 7.35 (d, 2H, Ar-H, *J* = 8.8 Hz); 7.52 (d, 2H, Ar-H, *J* = 9 Hz); 8.19 (d, 1H, CO-NH, *J* = 4.4 Hz); ^13^C-NMR (DMSO-*d_6_*): δ 172.55, 153.76, 148.30, 140.61, 140.57, 136.68, 135.89, 133.16, 120.25, 119.65, 115.66, 99.98, 75.28, 56.19, 31.29; MS (EI) *m/z*: 348 (M^+^, 7%); 163 (C_9_H_11_N_2_O, 100%).

*N-3-(2-Hydroxy-2-p-tolylethylamino)-N-methylbenzamide* (**19**). To a suspension of aminoketone **9**(0.5 g, 1.8 mmol) in methanol (10 mL) of at 15 °C, sodium borohydride (68.1 mg, 1.8 mmol) was added, then the mixture was allowed to stir at room temperature till complete dissolution. CH_2_Cl_2_ (30 mL) was then added and the resultant mixture was washed with 2 M NaOH solution (30 mL × 3). The combined aqueous layers were extracted with CH_2_Cl_2_ (30 mL), then the combined organic layers were dried (MgSO_4_), filtered and evaporated. The residue was crystallized from ethanol to yield 270.0 mg of **19 **(59.6%) as white crystals, mp: 137–139 °C; IR: 3,349, 3,299, 1,603, 1,583, 1,341, 1,060 cm^−1^; ^1^H-NMR (DMSO-*d_6_*): δ 2.28 (s, 3H, Ar-CH_3_); 2.73 (d, 3H, N-CH_3_, *J* = 4.4 Hz); 3.10 (m, 1H, CH_2_-N-Ar); 3.20 (m, 1H, CH_2_-N-Ar); 4.70 (m, 1H, Ar-CH); 5.39 (d, 1H, OH, *J* = 4 Hz); 5.65 (t, 1H, Ar-NH, *J* = 4.8, 6.8 Hz); 6.74 (dd, 1H, Ar-H, *J* = 1.6, 8 Hz); 6.96 (d, 1H, Ar-H, *J* = 7.8 Hz); 7.02 (s, 1H, Ar-H); 7.09 (d, 1H, Ar-H, *J* = 7.6 Hz); 7.14 (d, 2H, Ar-H, *J* = 8 Hz); 8.19 (d, 1H, CO-NH, *J* = 4.4 Hz); ^13^C-NMR (DMSO-*d_6_*): δ 167.79, 149.09, 141.59, 136.47, 135.84, 129.13, 129.04, 126.42, 115.53, 114.81, 110.82, 71.02, 51.80, 26.68, 21.18; MS (EI) *m/z*: 284 (M^+^, 11%); 163 (C_9_H_11_N_2_O, 100%).

*N-3-[2-(4-Chlorophenyl)-2-hydroxyethylamino]-N-methylbenzamide* (**20**). To a suspension of aminoketone **10**(0.5 g, 1.7 mmol) in methanol (10 mL) at 15 °C, sodium borohydride (64.3 mg, 1.7 mmol) was added, and then the mixture was allowed to stir at room temperature till complete dissolution. CH_2_Cl_2_ (30 mL) was then added and the resultant mixture was then washed with water (30 mL × 2) followed by 1 M NaOH solution (30 mL). The combined aqueous layers were extracted with CH_2_Cl_2_ (60 mL), and the combined organic layers were dried (MgSO_4_), filtered and evaporated. The residue was crystallized from ethyl acetate-ether to yield 0.3 g of **20** (53.6%) as white crystals, mp: 120–122 °C; IR: 3,445, 3,355, 2,904, 1,638, 1,342, 825 cm^−1^; ^1^H-NMR (DMSO-*d_6_*): δ 2.73 (d, 3H, N-CH_3_, *J* = 4.5 Hz); 3.14 (m, 1H, CH_2_-N-Ar); 3.22 (m, 1H, CH_2_-N-Ar); 4.74 (m, 1H, Ar-CH); 5.57 (d, 1H, OH, *J* = 4.4 Hz); 5.70 (t, 1H, Ar-NH, *J* = 5.8 Hz); 6.74 (dd, 1H, Ar-H, *J* = 1.7, 8 Hz); 6.96 (d, 1H, Ar-H, *J* = 7.8 Hz); 7.02 (s, 1H, Ar-H); 7.10 (t, 1H, Ar-H, *J* = 7.8 Hz); 7.40 (m, 4H, Ar-H); 8.19 (d, 1H, CO-NH, *J* = 4.5 Hz); ^13^C-NMR (DMSO-*d_6_*): δ 167.76, 148.99, 144.00, 135.85, 131.32, 129.14, 128.80, 120.44, 115.49, 114.87, 110.88, 70.55, 51.45, 26.68; MS (IR) *m/z*: 304 (M^+^, 11%); 163 (C_9_H_11_N_2_O, 100%).

*N-3-[2-Hydroxy-2-(4-iodophenyl)ethylamino]-N-methylbenzamide* (**21**). To a suspension of the aminoketone **11** (0.5 g, 1.3 mmol) in methanol (10 mL) at 15 °C, sodium borohydride (49.2 mg, 1.3 mmol) was added, then the mixture was allowed to stir at room temperature till complete dissolution. CH_2_Cl_2_ (50 mL) was then added and the resultant mixture washed with 0.5M NaOH solution (50 mL × 3). The combined aqueous layers were extracted with CH_2_Cl_2_ (50 mL), and the combined organic layers were dried (MgSO_4_), filtered and evaporated. The residue was crystallized from ethyl acetate-ether to yield 360.0 g of **21** (71.6 %) as white crystals; mp: 132–134 °C; IR: 3,352, 3,300, 1,603, 1,584, 1,342, 1,062 cm^−1^; ^1^H-NMR (DMSO-*d_6_*): δ 2.73 (d, 3H, N-CH_3_, *J* = 4.6 Hz); 3.12 (m, 1H, CH_2_-N-Ar); 3.21 (m, 1H, CH_2_-N-Ar); 4.70 (m, 1H, Ar-CH); 5.55 (d, 1H, OH, *J* = 4.4 Hz); 5.69 (t, 1H, Ar-NH, *J* = 5.9 Hz); 6.74 (dd, 1H, Ar-H, *J* = 1.6, 8.1 Hz); 6.96 (d, 1H, Ar-H, *J* = 7.6 Hz); 7.02 (s, 1H, Ar-H); 7.10 (t, 1H, Ar-H, *J* = 7.8 Hz); 7.21 (d, 2H, Ar-H, *J* = 8.2 Hz); 7.69 (d, 2H, Ar-H, *J* = 8.3 Hz); 8.19 (d, 1H, CO-NH, *J* = 4.4 Hz); ^13^C-NMR (DMSO-*d_6_*): δ 167.80, 148.99, 144.39, 137.19, 135.86, 135.82, 129.16, 128.98, 128.41, 115.50, 115.91, 110.91, 93.23, 70.66, 51.41, 26.70; MS (EI) *m/z*: 396 (M^+^, 11%); 163 (C_9_H_11_N_2_O, 100%).

*N-3-[2-(4-Acetylaminophenyl)-2-hydroxyethylamino]-N-methylbenzamide* (**22**). To a suspension of aminoketone **12** (2.5 g, 7.7 mmol) in CH_2_Cl_2_ (40 mL) and isopropanol (40 mL), sodium borohydride (351.8 mg, 9.3 mmol) was added and the reaction mixture was stirred overnight. Solvents were then evaporated and the residue taken up in ethyl acetate (50 mL) and washed with water (50 mL × 3). The organic layer was dried (MgSO_4_), filtered and evaporated. The residue was crystallized from absolute ethanol to yield 1.4 g of **22** (55.7%) as a white solid, mp: 177–179 °C; IR (KBr); 3,398, 3,307, 1,603, 1,545 cm^−1^; ^1^H-NMR (DMSO-*d_6_*): δ 2.02 (s, 3H, CH_3_-CO); 2.73 (d, 3H, N-CH_3_, *J* = 4.9 Hz); 3.11 (m, 1H, CH_2_-N-Ar); 3.21 (m, 1H, CH_2_-N-Ar); 4.68 (m, 1H, Ar-CH); 5.39 (d, 1H, OH, *J* = 4.2 Hz); 5.65 (t, 1H, Ar-NH, *J* = 5.7 Hz); 6.74 (dd, 1H, Ar-H, *J* = 1.3, 8.1 Hz); 6.96 (d, 1H, Ar-H, *J* = 7.6 Hz); 7.02 (s, 1H, Ar-H); 7.10 (t, 1H, Ar-H, *J* = 7.8 Hz); 7.30 (d, 2H, Ar-H, *J* = 8.4 Hz); 7.52 (d, 2H, Ar-H, *J* = 8.4 Hz); 8.19 (d, 1H, CO-NH, *J* = 4.6 Hz); ^13^C-NMR (DMSO-*d_6_*): δ 168.68, 167.83, 149.09, 139.15, 138.65, 135.83, 132.83, 129.15, 126.80, 119.20, 115.52, 114.82, 110.83, 70.90, 51.87, 26.68, 24.41; MS (EI) *m/z*: 327 (M^+^, 8%); 163 (C_9_H_11_N_2_O, 100%).

*N-3-[2-Hydroxy-2-(4-benzamidoaminophenyl)-ethylamino]-N-methylbenzamide* (**23**). To a suspension of aminoketone **13** (3.0 g, 7.5 mmol) in CH_2_Cl_2_ (80 mL) and isopropanol (80 mL), sodium borohydride (438.8 mg, 11.6 mmol) was added and the reaction mixture was stirred overnight. The suspension was then vacuum filtered, washed thoroughly with water then left to dry. The solid residue was crystallized from MeOH to yield 280.0 mg of **23** (9.3%) as a yellowish solid, mp: 189–191 °C; IR: 3,327, 1,651, 1,528, 1,322, 691 cm^−1^; ^1^H-NMR (DMSO-*d_6_*): δ 2.73 (d, 3H, N-CH_3_, *J* = 4.4 Hz); 3.14 (m, 1H, CH_2_-N-Ar); 3.24 (m, 1H, CH_2_-N-Ar); 4.73 (m, 1H, Ar-CH); 5.54 (bs, 1H, OH); 5.68 (t, 1H, Ar-NH, *J* = 5.8 Hz); 6.76 (dd, 1H, Ar-H, *J* = 2, 7.6 Hz); 6.97 (d, 1H, Ar-H, *J* = 7.6 Hz); 7.04 (s, 1H, Ar-H); 7.11 (t, 1H, Ar-H, *J* = 7.8 Hz); 7.37 (d, 2H, Ar-H, *J* = 8.4 Hz); 7.51 (m, 3H, Ar-H); 7.74 (d, 2H, Ar-H, *J* = 8.4 Hz); 7.95 (d, 2H, Ar-H, *J* = 7.2 Hz); 8.19 (d, 1H, CO-NH, *J* = 4.8 Hz); 10.22 (s, 1H, CO-NH-Ar); ^13^C-NMR (DMSO-*d_6_*): δ 176.82, 165.88, 149.11, 139.86, 138.44, 135.87, 135.38, 131.99, 129.15, 128.84, 128.08, 126.72, 120.52, 115.52, 114.85, 110.86, 70.87, 51.63, 26.68; MS (EI) *m/z*: 371 (M^+^-water, 2%); 227 (C_14_H_14_NO_2_, 34%).

*Phenyl* *α-chloroacetate* (**24**). A solution of phenol (5.0 g, 53.1 mmol) and pyridine (4.3 mL, 53.1 mmol) in CH_2_Cl_2_ (80 mL) was added dropwise to α-chloroacetyl chloride (8.5 mL, 106.9 mmol) and the mixture was allowed to stir vigorously for 2 h. The reaction mixture was then washed with water (200 mL × 2), 0.1 M NaOH solution (200 mL × 2) and 1 M HCl solution (100 mL). The organic layer was then dried (MgSO_4_), filtered then evaporated. The residue was crystallized from hexane to yield 6.2 g of **24** (68.1%) as white crystals, mp: 47–50 °C; IR: 3,068, 2,951, 1,773 cm^−1^; ^1^H-NMR (CDCl_3_): δ 4.47 (s, 2H, CO-CH_2_-Cl); 7.16 (d, 2H, Ar-H, *J* = 7.6 Hz); 7.30 (t, 1H, Ar-H, *J* = 7.4 Hz); 7.43 (t, 2H, Ar-H, *J* = 7.9 Hz); MS (EI) *m/z*: 170 (M^+^, 1%); 63 (COCl, 100%).

*Naphthalen-1-yl* *α-chloroacetate* (**25**). To a solution of 1-naphthol (10.0 g, 69.4 mmol) and α-chloroacetyl chloride (6.6 mL, 82.8 mmol) in CH_2_Cl_2_ (150 mL), triethyl amine (9.6 mL, 76.5 mmol) was added dropwise and the mixture was stirred for one hour. The reaction mixture was then washed with 1 M HCl solution (100 mL) followed by 1 M NaOH solution (100 mL × 3). The CH_2_Cl_2_ layer was then dried (MgSO_4_), filtered and evaporated. The residue was crystallized from ethyl acetate-hexane to yield 7.7 g of **25** (50.3%) as white crystals, mp: 113–115 °C; IR: 1,761, 1,162, 770 cm^−1^; ^1^H-NMR (CDCl_3_): δ 4.50 (s, 2H, CO-CH_2_-Cl); 7.32 (d, 1H, Ar-H, *J* = 7.6 Hz); 7.49 (t, 2H, Ar-H, *J* = 8 Hz); 7.55 (t, 1H, Ar-H, *J* = 4.6 Hz); 7.79 (d, 1H, Ar-H, *J* = 8 Hz); 7.91 (m, 2H, Ar-H); MS (EI) *m/z*: 220 (M^+^, 62%); 63 (COCl, 100%).

*Naphthalen-2-yl* *α-chloroacetate* (**26**). To a solution of 2-naphthol (10.0 g, 69.4 mmol) and α-chloroacetyl chloride (7.0 mL, 88.0 mmol) in CH_2_Cl_2_ (150 mL), triethyl amine (9.6 mL, 76.5 mmol) was added dropwise and the mixture was stirred for one hour. The reaction mixture was then washed with 1 M HCl solution (100 mL) and 1 M NaOH solution (100 mL × 3). The CH_2_Cl_2_ layer was then dried (MgSO_4_), filtered and evaporated. The residue was crystallized from ethyl acetate-hexane to yield 9.5 g of **26** (62.0%) as white crystals, mp: 99–102 °C; IR: 1,173, 1,163, 810 cm^−1^; ^1^H-NMR (CDCl_3_): δ 4.37 (s, 2H, CO-CH_2_-Cl); 7.27 (dd, 1H, Ar-H, *J* = 2, 8 Hz); 7.51 (m, 2H, Ar-H); 7.63 (d, 1H, Ar-H, *J* = 2 Hz); 7.86 (m, 3H, Ar-H); MS (EI) *m/z*: 220 (M^+^, 61%); 63 (COCl, 100%).

*N-Methyl-N-3-(1-phenoxyacetylamino)benzamide* (**27**). To a solution of aniline **1** (1.0 g, 6.7 mmol) and acetate **24** (1.1 g, 6.7 mmol) in DMF (17 mL), sodium bicarbonate (562.9 mg, 6.7 mmol) of and sodium iodide (1.4 g, 9.3 mmol) were added and the mixture was stirred overnight. The reaction mixture was then added to water (150 mL) and the precipitate was vacuum filtered and left to dry. The solid residue was crystallized from ethyl acetate-hexane to yield 320.0 mg of **27** (16.9%) as white crystals, mp: 137–139 °C; IR: 3,405, 3,320, 1,752 cm^−1^;^ 1^H-NMR (DMSO-*d_6_*): δ 2.75 (d, 3H, N-CH_3_, *J* = 4.5 Hz); 4.23 (d, 2H, CO-CH_2_-N-Ar, *J* = 6.4 Hz); 6.34 (t, 1H, Ar-NH, *J* = 6.4 Hz); 6.78 (dd, 1H, Ar-H, *J* = 2.2, 7.8 Hz); 7.05 (d, 1H, Ar-H, *J* = 7.6 Hz); 7.11 (d, 3H, Ar-H, *J* = 8.4 Hz); 7.17 (t, 1H, Ar-H, *J* = 7.8 Hz); 7.25 (t, 1H, Ar-H, *J* = 7.4 Hz); 7.42 (t, 2H, Ar-H, *J* = 7.8 Hz); 8.25 (d, 1H, CO-NH, *J* = 4.6 Hz); ^13^C-NMR (DMSO-*d_6_*): δ 170.75, 167.63, 150.82, 148.42, 135.88, 130.04, 129.25, 126.39, 122.11, 115.59, 115.38, 111.22, 45.22, 26.69; MS (EI) *m/z*: 284 (M^+^, 62%); 163 (C_9_H_11_N_2_O, 100%).

*N-Methyl-N-3-[1-(1-naphthoxy)acetylamino]-benzamide* (**28**). To a solution of the aniline **1** (1.0 g, 6.7 mmol) and acetate **25** (1.5 g, 6.7 mmol) in DMF (25 mL), sodium bicarbonate (0.6 g, 6.7 mmol) of and sodium iodide (1.5 g, 1.0 mmol) were added and the mixture was stirred for 4 h. The reaction mixture was then added to water (100 mL) and the precipitate was vacuum filtered and left to dry. The solid residue was crystallized from ethyl acetate-hexane to yield 783.0 mg (35.1%). IR (KBr): 3,397, 3,301, 1,758, cm^−1^; ^1^H-NMR (400 MHz, DMSO-*d*_6_): δ 2.8 (d, 3H, N-CH_3_, *J* = 3.8 Hz); 4.49 (d, 2H, CO-CH_2_-N-Ar, *J* = 6.6 Hz); 6.48 (t, 1H, Ar-NH, *J* = 5.4. 6.6 Hz); 6.89 (d, 1H, Ar-H, *J* = 8 Hz); 7.10 (d, 1H, Ar-H, *J* = 6.6 Hz); 7.22 (m, 2H, Ar-H); 7.32 (d, 1H, Ar-H, *J* = 7.5 Hz); 7.55 (m, 3H, Ar-H); 7.87 (t, 2H, Ar-H, *J* = 8.8, 10.9 Hz); 7.99 (d, 1H, Ar-H, *J* = 8.1 Hz); 8.31 (s, 1H, CO-NH). ^13^C-NMR (100 MHz, DMSO-*d_6_*): δ 171.12, 167.62, 148.51, 136.67, 135.92, 135.59, 129.32, 128.38, 127.12, 126.78, 126.46, 126.18, 121.73, 118.81, 115.70, 111.14, 45.20, 26.74. MS (EI) *m/z*: 334 (M^+^, 42%); 163 (C_9_H_11_N_2_O, 100%). mp: 164–166 °C.

*N-Methyl-N-3-[1-(2-naphthoxy)acetylamino]benzamide* (**29**). To a solution of the aniline **1**(1.0 g, 6.7 mmol) and acetate **26** (1.5 g, 6.7 mmol) in DMF (25 mL), sodium bicarbonate (562.9 mg, 6.7 mmol) of and sodium iodide (1.5 g, 1.0 mmol) were added and the mixture was stirred for 4 h. The reaction mixture was then added to water (100 mL) and the precipitate was vacuum filtered and left to dry. The solid residue was crystallized from ethyl acetate-hexane to yield 750.0 mg (33.7%) of **29**, mp: 150–152 °C; IR: 3,397, 3,312, 1,753, 1,644 cm^−1^; ^1^H-NMR (DMSO-*d_6_*): δ 2.77 (d, 3H, N-CH_3_, *J* = 4.4 Hz); 4.31 (d, 2H, CO-CH_2_-N-Ar, *J* = 6.6 Hz); 6.39 (t, 1H, Ar-NH, *J* = 6 Hz); 6.82 (dd, 1H, Ar-H, *J* = 1.5, 6.9 Hz); 7.07 (d, 1H, Ar-H, *J* = 5 Hz); 7.14 (s, 1H, Ar-H); 7.19 (t, 1H, Ar-H, *J* = 7.8 Hz); 7.30 (dd, 1H, Ar-H, *J* = 2.3, 8.8 Hz); 7.52 (dp, 2H, Ar-H, *J* = 1.6, 6.9 Hz); 7.68 (d, 1H, Ar-H, *J* = 2.3 Hz); 7.92 (m, 1H, Ar-H); 7.97 (d, 2H, Ar-H, *J* = 8.8 Hz); 8.27 (q, 1H, CO-NH, *J* = 4.4 Hz); ^13^C-NMR (DMSO-*d_6_*): δ 170.99, 167.65, 148.50, 135.90, 133.73, 131.47, 129.91, 129.30, 128.17, 127.92, 127.23, 126.35, 121.86, 118.89, 115.60, 111.28, 42.26, 26.72; MS (EI) *m/z*: 334 (M^+^, 63%); 144 (C_10_H_8_O, 100%).

*N-Phenyl* *α-chloroacetamide* (**30**). Aniline **1** (9.8 mL, 107.5 mmol) was dissolved in a biphasic mixture of ethyl acetate (220 mL) of and 1 M aqueous solution of sodium bicarbonate (129 mL). α-Chloroacetyl chloride (12.8 mL, 160.9 mmol) was then added dropwise to the biphasic mixture and the resultant mixture was stirred for one hour. The two layers were then separated and the aqueous layer extracted with ethyl acetate (100 mL). The combined organic layers were dried, filtered and evaporated to half the volume of ethyl acetate then allowed to cool to yield 15.0 g (82.3%) of grayish white crystals, mp: 140–143°C; ^1^H-NMR (CDCl_3_): δ 4.19 (s, 2H, CO-CH_2_-Cl); 7.18 (t, 1H, Ar-H, *J* = 7.4 Hz); 7.37 (t, 2H, Ar-H, *J =* 8 Hz); 7.55 (d, 2H, Ar-H, *J* = 7.6 Hz); 8.24 (bs, 1H, Ar-NH).

*N,N-Diphenyl* *α-chloroacetamide* (**31**). A solution of diphenylamine (5.0 g, 29.5 mmol) and triethyl-amine (4.1 mL, 29.5 mmol) in CH_2_Cl_2_ (70 mL) was added dropwise to a 2 M solution of α-chloroacetyl chloride (4.7 mL, 59.1 mmol) in CH_2_Cl_2_ and the mixture was stirred vigorously overnight. The reaction mixture was then washed with water (100 mL × 2) and 0.1N HCl solution (100 mL), dried (MgSO_4_), filtered then evaporated. The residue was crystallized from ethyl acetate to yield 4.2 g (58.2%) of **31**, mp: 124–127 °C; IR: 2,945, 1,682, 1,491, 1,265 cm^−1^; ^1^H-NMR (CDCl_3_): δ 4.03 (s, 2H, CO-CH_2_-Cl); 7.34 (m, 10H, Ar-H); ^13^C-NMR (CDCl_3_): δ 166.20, 141.81, 130.07, 129.118, 128.57, 126.66, 126.06; MS (EI) *m/z*: 245 (M^+^, 71%); 63 (COCl, 100%).

*N-Methyl 3-N,N-Di(phenylamidomethylamino)benzamide* (**32**). α-Chloroacetamide **30** (3.0 g, 17.7 mmol) and the aniline **1** (2.9 g, 19.3 mmol) were dissolved in DMF (40 mL). To that solution, sodium bicarbonate (1.5 g, 17.7 mmol) and sodium iodide (3.8 g, 25.4 mmol) were added and the mixture was stirred overnight. The reaction mixture was then added to water (300 mL) and the precipitate was vacuum filtered and left to dry. The solid residue was crystallized from ethanol to yield 2.0 g of **32** (54.3%, from **30** as the limiting reagent) as white crystals, mp: 248–250 °C; IR: 3,365, 3,300, 1,682, 1,660 cm^−1^; ^1^H-NMR (DMSO-*d_6_*): δ 2.72 (d, 3H, N-CH_3_, *J* = 4.4 Hz); δ 4.38 (s, 4H, CO-CH_2_-N-Ar); δ 6.67 (dd, 1H, Ar-H, *J* = 2.4, 8 Hz); δ 7.07 (m, 3H, Ar-H); δ 7.14 (d, 1H, Ar-H, *J* = 7.6 Hz); δ 7.27 (t, 2H, Ar-H, *J* = 8 Hz); δ 7.33 (t, 1H, Ar-H, *J* = 7.8 Hz); δ 7.65 (d, 4H, Ar-H, *J* = 8 Hz); δ 8.30 (d, 1H, CO-NH, *J* = 4.4 Hz), δ 10.88 (s, 2H, Ar-H); ^13^C-NMR (DMSO-*d_6_*): δ 170.36, 167.37, 147.42, 139.06, 136.09, 129.66, 129.40, 124.18, 119.67, 116.11, 114.12, 110.81, 57.30, 26.71; MS (EI) *m/z*: 296 (C_17_H_18_N_3_O_2_, 29%); 119 (C_7_H_6_NO, 9%).

*3-(Diphenylamidomethylamino)-N-methylbenzamide* (**33**). α-Chloroacetamide **31** (1.6 g, 6.7 mmol) and the aniline **1** (1.0 g, 6.7 mmol) were dissolved in DMF (40 mL). To that solution, sodium carbonate (562.9 mg, 6.7 mmol) and sodium iodide (1.5 g, 1.0 mmol) were added and the mixture was stirred overnight. The reaction mixture was then added to water (200 mL) and the precipitate was vacuum filtered and left to dry. The solid residue was crystallized from ethyl acetate to yield 1.2 g (50.1%) of **33** as white crystals, mp: 171–173 °C; IR): 3,449, 3,373, 1,684, 1,505, 1,294 cm^−1^; ^1^H-NMR (DMSO-*d_6_*): δ 2.74 (d, 3H, N-CH_3_, *J* = 4.5 Hz); δ 3.72 (d, 2H, CO-CH_2_-N-Ar, *J* = 5.7 Hz); δ 5.94 (t, 1H, Ar-NH, *J* = 5.8 Hz); δ 6.64 (dd, 1H, Ar-H, *J* = 1.8, 8 Hz); δ 6.93 (s, 1H, Ar-H); δ 6.99 (d, 1H, Ar-H, *J* = 7.9 Hz); δ 7.11 (t, 1H, Ar-H, *J* = 7.8 Hz); δ 7.43 (m, 10H, Ar-H); δ 8.2 (d, 1H, CO-NH, *J* = 4.5 Hz); ^13^C-NMR (DMSO-*d_6_*): δ 169.93, 167.76, 148.50, 142.65, 135.81, 129.81, 129.13, 127.24, 118.77, 115.40, 115.11, 111.33, 46.22, 26.72 MS (EI) *m/z*: 359 (M^+^, 73%); 163 (C_9_H_11_N_2_O, 100%).

### 3.3. Molecular Modeling

The chemical structure of 2-aminoalcohol **16** was drawn in ChemDraw Ultra v10 as a chm file and was then opened by Hyperchem 7.5. Using the “build” command, a three-dimensional conformation was obtained and the partial atomic charges were calculated using AM1 semi-empirical calculations. The obtained structure was then geometry optimized using Enhanced MM (MM+) force field implemented in HyperChem using the atomic charges and Polak-Ribiere conjugate gradient with an RMS gradient of 0.01 kcal/Ǻ mol options. For the computation of intramolecular hydrogen bonding, *Ab initio* geometry optimization for the most stable conformer obtained by MM+ using Polak-Ribiere conjugate gradient with an RMS gradient of 0.01 kcal/Ǻ mol was performed.

## 4. Conclusions

Ten α-anilinoketones, eight 2-aminoalcohols, three α-anilinoesters and two α-anilinoamides were successfully synthesized and characterized by NMR spectroscopy and mass spectrometry. The yields were moderate to good for the α-anilinoketones (up to 65.4%) and the 2-aminoalcohols (up to 75.4%), low for the α-anilinoesters (16.8–35.1%) and low to moderate for the α-anilinoamides (50.1–54.3%). In addition, α-haloketones and α-chloroacetates afforded monoalkylated products when reacted with 3-amino-*N*-methylbenzamide (**1**), while small α-chloroamides afforded the dialkylated product. This implied that the mechanisms for the nucleophilic substitution reactions are not similar and that alkylation by α-chloroamides, only, proceeds by direct S_N_2 mechanism. ^1^H-NMR spectroscopy showed that the chemical structure of the 2-aminoalcohols is rigidified by the possible formation of a 5-membered ring due to an intramolecular hydrogen bond, while ^13^C-NMR spectroscopy indicates that diphenylamides contain two nonequivalent, but not totally distinct phenyl groups that give rise to broad unresolved peaks.

## References

[1] Ando R., Kawamura M. (2001). *N*-3-(Arylacetylamino)-*N*-methylbenzamide: A novel class of selective anti-*Helicobacter pylori* agents. J. Med. Chem..

[2] Ando R., Kawamura M., Chiba N., Watanabe K. (2002). Amide derivatives. US Patent.

[3] Parsonnet J. (1998). *Helicobacter* *pylori*. Infect. Dis. Clin. N. Am..

[4] Kuipers E., Thijs J., Festen H. (1995). The prevalence of *Helicobacter pylori* in peptic ulcer disease. Aliment. Pharmacol. Ther..

[5] Graham D.Y., Yamaoka Y. (1998). *H. pylori* and cagA: Relationships with gastric cancer, duodenal ulcer, and reflux esophagitis and its complications. Helicobacter.

[6] Parkin D.M., Pisani P., Ferlay J. (1993). Estimates of the worldwide incidence frequency of eighteen major cancers in 1985. Int. J. Cancer.

[7] Xia H.H.-X., Talley N.J. (2001). Apoptosis in gastric epithelium induced by *Helicobacter pylori* infection: Implications in gastric carcinogenesis. Am. J. Gastroenterol..

[8] Heatley R. (1998). The Helicobacter pylori Handbook.

[9] Murakami K., Fujioka T., Nishizono A., Nagai J., Tokeida M., Kodama R., Kubota T., Nasin M. (1996). Atopic dermatitis successfully treated by eradication of *Helicobacter pylori*. J. Gastroenterol..

[10] Gubbins G.P., Moritz T.E., Marsano L.S., Talwalker R., McClain C.J., Mendenhall C.L. (1993). *Helicobacter pylori* is a risk factor for hepatic encephalopathy in acute alcoholic hepatitis: The ammonia hypothesis revisited. The Veterans Administration Cooperative Study Group No. 275. Am. J. Gastroenterol..

[11] Pasceri V., Cammarota G., Patti G., Cuoco L., Gasbarrini A., Grillo R.L., Fedeli G., Gasbarrini G., Maseri A. (1998). Association of virulent *Helicobacter pylori* strains with ischemic heart disease. Circulation.

[12] Vogel A. (1956). A Textbook of Practical Organic Chemistry Including Qualitative Organic Analysis.

[13] Klingenberg J. (1950–1959). *p*-Bromomandelic acid. Organic Synthese.

[14] Leiserson J., Weissberger A. (1940–1949). *p*-Chloroacetylacetanilide. Organic Synthese.

[15] Loeser E., Prasad K., Repic O. (2002). Selective *N*-alkylation of primary amines with chloroacetamides under pH-controlled aqueous conditions. Synth. Commun..

[16] Jensen A., Kneisel F., Knochel P. (1998–2002). Ethyl 3-(*p*-cyanophenyl)propionate from ethyl 3-iodopropionate and *p-*cycanophenylzinc bromide.

[17] Erian A., Sherif S., Gaber H. (2003). The chemistry of a-haloketones and their utility in heterocyclic synthesis. Molecules.

[18] van-Vliet L., Rodenhuis N., Dijkstra D., Wikstrom H., Pugsley T., Serpa K., Meltzer L., Heffner T., Wise L., Lajiness M. (2000). Synthesis and pharmacological evaluation of thiopyran analogues of the dopamine D3 receptor-selective agonist (4aR,10bR)-(+)-trans-3,4,4a,10b-tetrahydro-4-n-propyl-2H,5H [1]b enzopyrano[4,3-b]-1,4-oxazin-9-ol (PD 128907). J. Med. Chem..

[19] Dilling W.L., Plepys R. (1970). Metal hydride reductions of *endo*-tricyclo[5.2.1.0^2,6^]deca-4,8-dien-3-one-(endo-dicyclopentadienone). J. Org. Chem..

[20] Mehta C., Murthy A.N., Reddy D.S., Reddy A.V. (1986). A General Approach to Linearly Fused Triquinane Natural Products. Total Syntheses of (±)-Hirsutene, (±)-Coriolin, and (±)-Capnellene. J. Am. Chem. Soc..

[21] Gogoll A., Grennberg H., Axen A. (1997). Chemical shift assignment of geminal protons in 3,7-diazabicyclo[3.3.1]nonanes: An unexpected deviation from the axial/equatorial chemical shift order. Magn. Reson. Chem..

[22] Cadet J., Taieb C., Remin M., Niemczura W.P., Hruska F.E. (1980). Conformational studies of a- and b-pyridine 2'-deoxyribonucleosides in the *syn* and *anti* conformation. Biochi. Biophys. Acta.

[23] Kover K.E., Feher K. (2004). Measurement of one-bond heteronuclear dipolar coupling contributions for amine and diastereotopic methylene protons. J. Magn. Reson..

[24] Kazlauskas R.J. (1992–1997). (S)-(−)-and (R)-(+)-1,1'-Bi-2-Naphthol. Organic Synthese.

[25] Alali F.Q., Ma'aya'h A.S., Alkofahi A., Qandil A., Li C., Burgess J., Wani M.C., Oberlies N.H. (2006). A New colchicinoid from *Colchicum tauri*, an unexplored meadow saffron native to Jordan. Nat. Prod. Commun..

[26] Chemexper.com 2-Bromo-1-indanone, 1775-27-5. http://www.chemexper.com/.

[27] Kajigaeshi S., Kakinami T., Moriwaki M., Fujisaki S., Maeno K., Okamoto T. (1988). α-Chlorination of aromatic acetyl derivatives with benzyltrimethylammonium dichloroiodate. Synthesis.

